# Augmented concentrations of CX_3_CL1 are associated with interstitial lung disease in systemic sclerosis

**DOI:** 10.1371/journal.pone.0206545

**Published:** 2018-11-20

**Authors:** Anna-Maria Hoffmann-Vold, Stephen Samuel Weigt, Vyacheslav Palchevskiy, Elizabeth Volkmann, Rajan Saggar, Ning Li, Øyvind Midtvedt, May Brit Lund, Torhild Garen, Michael C. Fishbein, Abbas Ardehali, David J. Ross, Thor Ueland, Pål Aukrust, Joseph P. Lynch, Robert M. Elashoff, Øyvind Molberg, John A. Belperio

**Affiliations:** 1 Department of Rheumatology, Oslo University Hospital, Rikshospitalet, Oslo, Norway; 2 Institute of Clinical Medicine, University of Oslo, Rikshospitalet, Oslo, Norway; 3 Department of Medicine, David Geffen School of Medicine at UCLA, Los Angeles, California, United States of America; 4 Department of Biomathematics, David Geffen School of Medicine at UCLA, Los Angeles, California, United States of America; 5 Department of Pulmonary Disease, Oslo University Hospital, Rikshospitalet, Norway; 6 Department of Pathology, UCLA, Los Angeles, California, United States of America; 7 Department of Surgery, UCLA, Los Angeles, California, United States of America; 8 Section of Clinical Immunology and Infectious Diseases, Oslo University Hospital, Rikshospitalet, Norway; 9 K.G. Jebsen Inflammatory Research Center, University of Oslo, Norway; 10 Research Institute of Internal Medicine, Oslo University Hospital, Rikshospitalet, Oslo, Norway; Keio University, JAPAN

## Abstract

**Background:**

Dysregulation of Fractalkine (CX_3_CL1) and its receptor CX_3_CR1 has been linked to the pathobiology of chronic inflammatory conditions. We explored CX_3_CL1 in systemic sclerosis (SSc) related progressive interstitial lung disease (ILD) and pulmonary hypertension (PH) in two different but complementary sources of biomaterial.

**Methods:**

We collected lung tissue at the time of lung transplantation at UCLA from SSc-ILD patients (n = 12) and healthy donors (n = 12); and serum samples from the prospective Oslo University Hospital SSc cohort (n = 292) and healthy donors (n = 100). CX_3_CL1 was measured by ELISA. Cellular sources of CX_3_CL1/CX_3_CR1 in lung tissues were determined by immunohistochemistry and immunofluorescence. ILD progression and new onset PH endpoints were analysed.

**Results:**

CX_3_CL1 concentrations were increased in SSc in lung tissue as well as in sera. In the UCLA cohort, CX_3_CL1 was highly correlated with DLCO. In the SSc-ILD lungs, CX_3_CL1 was identified in reactive type II pneumocytes and airway epithelial cells. CX_3_CR1 stained infiltrating interstitial mononuclear cells, especially plasma cells. In the Oslo cohort, CX_3_CL1 correlated with anti-Topoisomerase-I-antibody and lung fibrosis. CX_3_CL1 was associated with ILD progression in multivariable regression analysis but not PH.

**Conclusion:**

CX_3_CL1 is associated with progressive SSc-ILD but not SSc-PH. The CX_3_CR1/CX_3_CL1-biological axis may be involved in recruiting antibody secreting plasma cells to SSc lungs, thereby contributing to the immune-mediated pathobiology of SSc-ILD.

## Introduction

Systemic sclerosis (SSc) is a connective tissue disease characterized by distinct serum auto-antibodies, vascular remodelling and chronic inflammation that drives fibrotic processes in multiple organs [1–3]. With the onset of therapeutic strategies against rapidly progressive renal vascular damage (scleroderma renal crisis), survival among patients with SSc is predominately influenced by the development of pulmonary manifestations, more specifically interstitial lung disease (ILD) and pulmonary hypertension (PH) [4–6]. Multiple studies have demonstrated that the risk of death in SSc patients with these pulmonary complications increases 2 to 7 times [5–6]. The risk for ILD and PH is evident in SSc patients with the limited cutaneous form of the disease (lcSSc), where skin involvement is only detectable in areas distal to elbows and knees, and in those with the diffuse cutaneous form (dcSSc), where thickening of the skin occurs both proximal and distal [1]. The presence of anti-centromere antibodies (ACA) has been associated with PH while anti-topoisomerase I antibodies (ATA) associate with ILD, but these antibodies have poor negative predictive value [1,7].

The clinical course of PH and ILD in SSc is highly variable ranging from slowly evolving cases that may respond to targeted therapy to more progressive forms that may result in right heart failure or pulmonary fibrosis with respiratory failure and death [7,8]. The pathological processes leading to SSc-PH and SSc-ILD are not well understood, but they most likely involve complex mechanisms driven by inappropriate immune activation and enhanced fibrogenesis in the lungs, but the molecules that drives these interacting processes are not fully understood and hamper targeted therapeutic intervention [2,9]. Thus, an understanding of the pathobiology involved in SSc-PH and SSc-ILD could potentially lead to novel pharmacological targets that may either reverse or prevent the progression of these SSc associated pulmonary complications.

CX_3_CL1 is the only member of the CX_3_C chemokine subfamily and can function both as a chemoattractant and an adhesion molecule where the membrane-anchored protein is expressed primarily on the inflamed endothelium, promoting the retention of monocytes and T cells, whereas the soluble form resembles more a conventional chemokine and strongly induces chemotaxis. Both chemotaxis and adhesion are mediated by CX_3_CR1, a seven transmembrane G-protein coupled receptor that is expressed predominately on mononuclear cells [10,11]. Based on CX_3_CL1 association with WHO group 1 PAH [12], its injurious role described in rodent bleomycin induced acute lung injury and pulmonary fibrosis, and its strong association with other chronic inflammatory fibroproliferative diseases such as rheumatoid arthritis, Sjogren’s disease, and systemic lupus erythematosus [13], we hypothesized that the CX_3_CR1/CX_3_CL1 axis may be involved in the pathogenesis of SSc associated pulmonary diseases. More specifically, we determined the concentrations of CX_3_CL1 in whole lung homogenates from patients with pulmonary SSc-ILD with or without PH compared to normal lung tissue. Moreover, we explored the association between serum concentrations of CX_3_CL1 and SSc associated pulmonary diseases and their severity.

## Materials and methods

### UCLA SSc study cohort

This study was approved by the Institutional Review Board in Los Angeles, CA, USA, (#16–000117). Written consent was obtained of all included patients after verbal and written information. All SSc cases in the UCLA cohort (n = 12) met the 2013 EULAR/ACR classification criteria for SSc, had available pulmonary function tests (PFTs), chest high resolution CT scans (HRCT), and lung explant tissue samples available for CX_3_CL1 analyses. Clinical and demographic data, antibodies, vital status and treatment were collected by retrospective chart review. Immune modulating treatment included the use of cyclophosphamide, mycofenolate mofetil, azathioprine, rituxan, hydroxychloroquine and prednisone. All lung material was collected from the left lung and sampled at the interface between severely fibrotic lung and less involved lung tissue. The control group consisted of 12 biopsies from normal donor lungs being used for lung transplantation. Donor biopsies were done after harvesting and just prior to implantation in the recipient. Control lungs had a cold-ischemia time less than 4 hours and all were assessed as having no significant post-transplant ischemia reperfusion injury (i.e. primary graft dysfunction grade 0).

### OUH study cohort and clinical parameters

The OUH portion of this study was approved by the regional committee of health and medical research ethics in South-East Norway (No. 2003–01787). Written consent was obtained of all included patients after verbal and written information. All of the OUH SSc patients were included in an ongoing, prospective, observational SSc cohort and met the 2013 EULAR/ACR classification criteria for SSc and had sera for CX_3_CL1 analyses available [14–16]. Clinical and demographic data (including the modified Rodnan Skin Score (mRSS), SSc antibodies,vital status and immune modulating treatment) were obtained and subsets were defined as lcSSc and dcSSc [17]. Disease onset of SSc was defined as the first non-Raynaud symptom. Time from disease onset to study end (June 2017) or time of death was defined as follow up period. Healthy controls were drawn from the blood bank for blood donors at OUH. The Norwegian law for blood donor is strict, allowing only inclusion of healthy individuals without cardiovascular disease, immune deficiencies, any chronic diseases and infections.

### Serial assessment of ILD for OUH and UCLA cohorts

PFTs and HRCT lung images were obtained at baseline and at last available follow-up visit and extent of fibrosis was measured as previously described [14]. Briefly, reticular pattern abnormalities and super-imposed ground-glass opacities defined as equivalent to fibrosis were measured precisely by freehand drawing of the region of interest on CT images reconstructed at 1.25 mm section thickness in 10 mm intervals in 10 images per lung [18]. Pulmonary fibrosis was expressed as percentage of total lung volumes; an extent of >0.1–10% pulmonary fibrosis was considered as mild ILD and an extent of pulmonary fibrosis ≥10% was defined as clinically significant ILD [19]. Annual fibrosis progression was defined as the difference in extent of fibrosis between the baseline and follow up HRCTs divided by the actual follow-up period (in years) and an annual fibrosis progression ≥ 5% was considered as clinical significant (14). PFTs were carried out according to ATS-ERS guidelines using automated Vmax V6200 (SensorMedics) [20]. Recorded PFT variables were: forced vital capacity (FVC) and forced expiratory volume in 1 second (FEV1). Gas diffusing variables were the transfer factor for carbon monoxide (DLCO) and DLCO divided by alveolar volume (DLCO/VA). PFT values were expressed in absolute values and as percentage of predicted values. Annual DLCO and/or FVC decline was defined as the difference of DLCO% and/or FVC% predicted between baseline and follow-up divided by the actual follow-up period in years. Annual DLCO decline >7.5% and annual FVC decline >5% was defined as clinically significant [14,21]. Primary endpoint was an ILD progression free survival composite outcome. Events for ILD progression included (I) annual DLCO decline >7.5%, (II) annual FVC decline >5%, or (III) all-cause mortality within 12 months after sera sampling. Secondary endpoints included (I) new onset of significant ILD on HRCT during the follow up (progression of lung fibrosis from < 10% to >10% fibrosis on HRCT), and (II) annual progression of extent of pulmonary fibrosis ≥5%.

### Assessment of pulmonary hypertension

All transplanted SSc patients from the UCLA cohort underwent intraoperative trans-esophageal echocardiography and Swan-Ganz catheter hemodynamic assessment at the time of lung transplant. In the OUH SSc cohort, longitudinal paired NT-pro BNP data and systolic pulmonary artery pressure (sPAP) measured by ECHO were obtained both at baseline and at the last available follow-up visit. Indication for right heart catheterization (RHC) was PH suspicion because of clinical symptoms, increasing sPAP on ECHO, increasing NT-pro BNP, and declining DLCO. PH was diagnosed by an experienced cardiologist or pulmonologist according to the updated European Society of Cardiology guidelines with a mean pulmonary arterial pressure (mPAP) ≥ 25 mmHg measured by RHC, further divided into pre- and post-capillary PH according to a pulmonary wedge pressure ≤ 15 mmHg or > 15 mmHg, respectively [22]. WHO group 1 PAH was defined as the presence of precapillary PH and the absence of significant lung fibrosis with <10% lung fibrosis on HRCT and FVC% predicted > 70% at both baseline and follow up; with other causes of precapillary PH ruled out [19,23]. WHO group 3 PH (PH-ILD) consisted of patients with precapillary PH and findings of significant pulmonary fibrosis involving >10% lung fibrosis on HRCT and FVC% predicted ≤ 70%. Primary endpoint was a PH free outcome. New onset of PH diagnosed by RHC during the observation period was considered as a PH event. Association between CX_3_CL1 and new onset PH were conducted including all PH cases with blood samples taken at least 6 months prior to RHC. Secondary endpoints were (I) new onset of sPAP on ECHO ≥30mmHg and (II) increase of sPAP on ECHO ≥10mmHg and (III) increase of pro- NT-BNP >165 pg/mL during the follow up period.

### Sample processing and CX_3_CL1 measurement

Explanted SSc and donor lungs were immediately placed in media and then on ice and transferred to the laboratory where they were homogenized and sonicated in anti-protease buffer using a method as previously described [24]. Specimens were centrifuged at 900*g* for 15 min, filtered through a 1.2 μm sterile Acrodiscs (Gelman Sciences, Ann Arbor, MI), and frozen at −70°C until thawed for CX_3_CL1 concentrations. Protein concentrations of CX_3_CL1 in lung homogenates were performed with by enzyme immunoassay (R&D Systems, Stillwater, MN, USA).

Blood from the OUH SSc cohort was centrifuged at room temperature within 30 min and serum aliquots were stored at –70°C until assayed. Serum samples from 100 healthy individuals drawn at random from the OUH blood bank served as controls. CX_3_CL1 was analyzed by enzyme immunoassay (R&D Systems, Stillwater, MN, USA) using undiluted serum according to manufacturer instructions.

### Immunohistochemistry of CX_3_CL1 and CX_3_CR1

Immunohistochemistry staining was performed on paraffin-embedded samples of 5 lung tissues from SSc-ILD patients using the VECTASTAIN ABC Elite kits (Vector Laboratories, Inc., Burlingame, CA, USA) as previously described [25,26]. After deparaffinization, rehydration and antigen retrieval in sodium citrate buffer (pH 6.0), endogenous perioxidase was quenched with 3% hydrogen peroxide. Sections were incubated in appropriate blocking serum to minimize non-specific binding and endogenous biotin was blocked with an avidin/biotin blocking kit (Vector Laboratories). Slides were incubated overnight with primary antibody at 4°C. The primary antibodies for rabbit polyclonal anti-human CX_3_CL1 (PA5 23062) and rabbit polyclonal anti-human CX_3_CR1 (PA5 32713) were purchased from Thermo-Fischer Scientific (Grand Island, NY, USA). Primary antibody for goat polyclonal anti-human CD138 (AF 2780) was purchased from R&D systems. Specific labeling was detected with a species specific biotinylated secondary antibody and application of horseradish peroxidase-conjugated avidin-biotin followed by development with 3,3′-diaminobenzidine (DAB) solution (Vector Laboratories). Stained slides were counterstained with hematoxylin, mounted and analyzed for cellular sources of specific chemokines and their receptors.

### Immunostaining of CX_3_CR1 and CD138

Human autopsy tissues of lung tissues from SSc-ILD patients were fixed in 4% paraformaldehyde and paraffin-embedded. For immunofluorescence staining, four-micrometer sections were blocked with 10% normal donkey solution and stained with primary antibodies: rabbit anti-CX3CR1 (Thermo Fisher Scientific, Grand Island, NY, USA, 1:1000, cat no. PA5-32713) and goat anti-Syndecan 1/CD138 (R&D Systems Inc. Minneapolis, MN, USA, 1:1000, cat no. AF2780). Sections were subsequently stained with anti-goat Alexa Fluor 594 (cat no. A-11058) and anti-rabbit Alexa Fluor 488 (cat no. A-21206) fluorophore-conjugated secondary antibodies (Thermo Fisher Scientific, Grand Island, NY, USA; 1:1000). Slides were mounted with Fluoromount G containing 4′-6-diamidino-2-phenylindole (DAPI). Stained slides were digitally scanned by Translational Pathology Core Laboratory (UCLA, Los Angeles, CA, USA) using Leica Versa high-throughput scanning system and analyzed on Aperio ImageScope software (Leica Biosystems Inc., Buffalo Grove, IL USA).

### Statistical analyses

Analyses were performed by SPSS version 25 and STATA version 14. Pearson Chi-square test, Fishers exact test and Kruskal-Wallis t-test were used as appropriate. For correlations analyses, Pearson or Kendall’s tau-b coefficients were applied as appropriate. Logistic regression analyses with Odds ratio (OR) with its 95% confidence interval (CI) were applied to analyse primary endpoints (PH free endpoint and ILD progression free survival composite endpoint) and secondary endpoints. Multivariable analyses were preceded by estimation of correlation between risk factors. For both outcome measures independent risk factors from univariable analyses, at a significance level of 20%, were included in the multivariable logistic regression analysis. A backward stepwise elimination procedure was performed to identify independent risk factors. The final model was evaluated by the area under the Receiver operating characteristic (ROC) curve (AUC) (values>0.7 were considered as acceptable).

## Results

### UCLA SSc cohort demographic and clinical characteristics at the time of lung transplant

The UCLA SSc cohort age was 39.3 years (SD 8.1), 58.3% were males, 25% were Caucasian, 33.3% Black, and 41.7% Hispanic (Table 1). Three patients (25%) had dcSSc, 37.5% were ATA positive and 75% had GI dysmotility (Table 1). All patients were on immune modulating treatment at the time of lung transplantation.

**Table 1:** Demographics and clinical characteristics from the Oslo University Hospital and the University of California at Los Angeles.

Clinical characteristics	OUH cohort (n = 292)	UCLA cohort (n = 12)
Age at onset, yrs mean (SD)	48 (15.4)	39.3 (8.1)
Follow-up period, yrs mean (SD)	11.5 (8.1)	13.3 (7.5)
Ethnicity, n (%)		
White	275 (94.2)	3 (25)
Black	2 (0.7)	4 (33.3)
Hispanic	0	5 (41.7)
Asian	15 (5.1)	0
Male, n (%)	53 (18.2)	7 (58.3)
dcSSc, n (%)	77 (26.4)	3 (25)
Deceased, n (%)	85 (29.1)	5 (41.7)
ATA, n (%)	47 (16.1)	3 (37.5)
ACA, n (%)	124 (42.5)	2 (16.7)
PH, n (%)	65 (22.3)	8 (66.7)
PAH	40 (13.7)	0
PH-ILD	25 (8.6)	8 (66.7)
Digital ulcers, n (%)	140 (47.9)	6 (50)
GAVE, n (%)	23 (7.9)	0
SRC, n (%)	9 (3.1)	0
Dysphagia, n (%)	145 (49.7)	10 (83.3)
Esophagus dysmotility, n (%)	215 (73.6)	9 (75)
mRSS	9.4 (9.1)	3.8 (3.1)
**Lung characteristics**		
Baseline FVC, % mean (SD)	94.7 (20.3)	50.3 (14.7)
Baseline DLCO, % mean (SD)	66.4 (21.7)	32.4 (14.6)
Baseline fibrosis, % mean (SD)	6.3 (11.7)	n.a.
Baseline sPAP, mmHg	27.3 (19.3)	n.a.
Baseline NT proBNP, pmol/L	74.3 (307.5)	n.a.
Baseline 6MWD, m mean (SD)	430 (185.9)	335 (151.2)
mPAP at diagnosis*, mmHg	30.9 (12.6)	39.4 (15.1)
Follow-up* FVC, % mean (SD)	90.3 (22.5)	42.9 (10.5)
Follow-up* DLCO, % mean (SD)	59.9 (20.9)	20.6 (5.1)
Follow-up* fibrosis, % mean (SD)	8.2 (14.5)	55.1 (11.6)
Follow-up* sPAP, mmHg	34.7 (23.7)	n.a.
Follow-up* NT proBNP, pmol/L	201.9 (582.5)	n.a.

OUH: Oslo University Hospital; UCLA: University of California Los Angeles; No: number; SD: standard deviation; SSc: systemic sclerosis; dcSSc: diffuse cutaneous systemic sclerosis; ATA: ant-topoisomerase I antibody; ACA: anti centromere antibody; PH: pulmonary hypertension; PAH: pulmonary arterial hypertension; GAVE: Gastric Antral Vascular Ectasia; SRC scleroderma renal crisis; FVC: forced vital capacity; DLCO: diffusing Lung capacity for carbon monoxide; n.a.: not available; sPAP: systolic pulmonary arterial pressure measured on ECHO; NTpro-BNP: N-terminal pro-brain natriuretic peptide; m: meters; mPAP: mean pulmonary artery pressure by right heart catheterization; 6MWD: six-minute-walking-distance

* In UCLA cohort time of transplant

All 12 SSc patients had clinically significant ILD with 55.1% pulmonary fibrosis on HRCT, FVC 42.9% and DLCO 20.6% at time of lung transplant (Table 1). All patients had a marked annual decline in lung function before lung transplant as seen with a reduction in FVC of 7.7% and/or DLCO 19.3% from baseline values prior to lung transplant. At the time of transplant, 70% of the SSc patients had PH diagnosed by RHC with a mean PAP of 39.4 mmHg (Table 1).

### CX_3_CL1 concentrations are increased in lung homogenates from patients with SSc associated ILD

**Fig 1:**
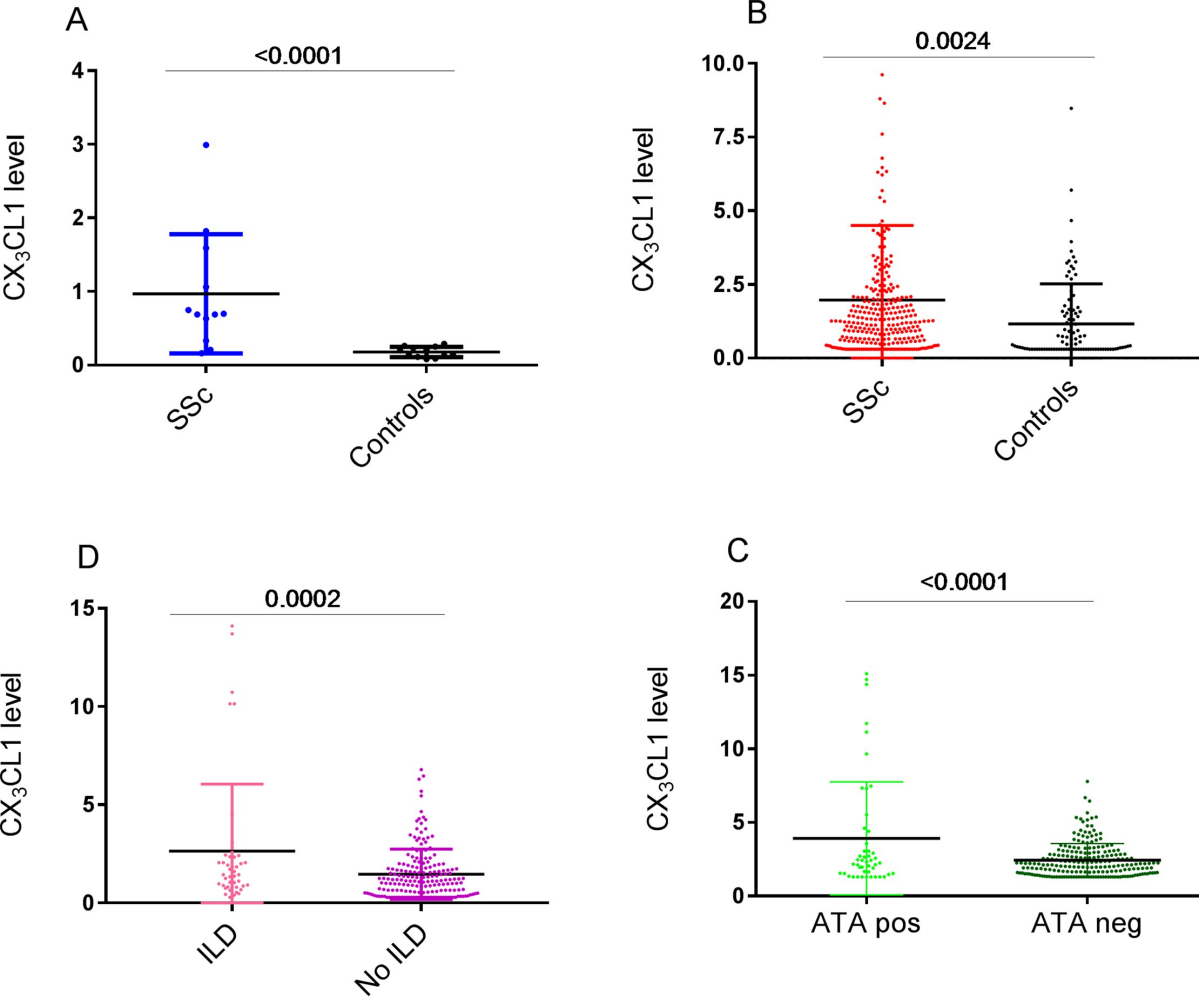
Augmented CX_3_CL1 concentrations in SSc associated ILD. (A) CX_3_CL1 protein concentration in fresh explanted lung tissue homogenates from the UCLA SSc-ILD cohort (n = 12) with and without PH, as compared to healthy lung tissue homogenate control tissue from donor lungs that did not develop any PGD (n = 12). (B) Serum concentrations of CX_3_CL1 from patients with SSc from the OUH cohort (n = 292) as compared to healthy controls without any systemic diseases (n = 100). (C) Serum concentrations of CX_3_CL1 in the OUH SSc cohort from patients with (n = 51) and without (n = 241) SSc-ILD at baseline. (D) Serum concentrations of CX_3_CL1 in the OUH SSc cohort from patients with (n = 47) and without (n = 245) a positive ATA.

CX_3_CL1 concentration in whole lung homogenates was significantly increased from the SSc lungs (0.97 ng/ml, (SD 0.80) compared to healthy lung donors 0.18 ng/ml (SD 0.06), p<0.001) (Fig 1). CX_3_CL1 correlated significantly with DLCO% predicted at time of transplant (r = 0.9, p = 0.036). There was no significant association between CX_3_CL1 and PH, and no significant association between CX_3_CL1 levels and male gender, dcSSc, mortality, modified Rodnan skin score (mRSS) or gastrointestinal symptoms.

### CX_3_CL1 is expressed by epithelial cells and mononuclear cells whereas its receptor CX_3_CR1 is predominately expressed by mononuclear cells including plasma cells

**Fig 2:**
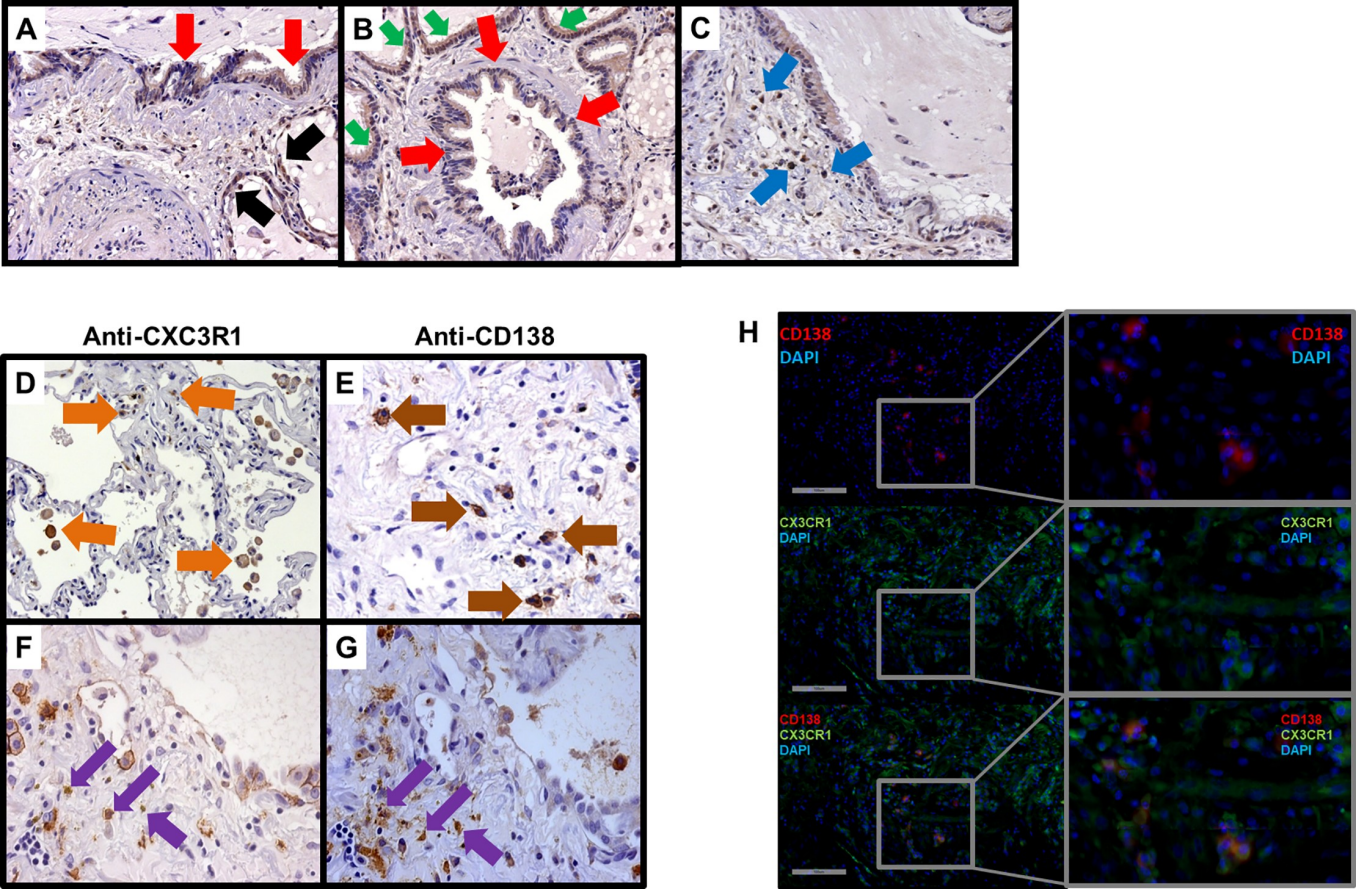
Immunostaining of CX_3_CL1 and CX_3_CR1 in SSc-ILD. CX_3_CL1 protein is produced from the epithelium and infiltrating interstitial leukocytes in SSc-ILD, while its receptor CX_3_CR1 is localized to infiltrating mononuclear cells. Representative histopathological staining of (n = 5) SSc-ILD for CX_3_CL1 from (A) Type 2 pneumocytes, (B) Airway epithelium, (C) Epithelium involved in bronchiolization, and (D) Infiltrating mononuclear cells. Representative histopathological staining of (n = 5) SSc-ILD for CX_3_CR1 from (E) Infiltrating interstitial mononuclear cells, (F) Morphologically from plasma cells via their eccentric cartwheel nuclei, (G) Representative staining of plasma cell marker CD138 confirming that the cells with eccentric cartwheel nuclei are plasma cells, (H) Representative immunofluorescence staining of plasma cell (red) in the interstitium, CX_3_CR1 positive infiltrating mononuclear cells, and co-localization of CD138^+^ plasma cell expressing CX_3_CR1 and infiltrating the interstitium.

Using immunohistochemistry (IHC) techniques we determined the cellular sources of CX_3_CL1 from the lungs of the SSc patients (n = 5). IHC demonstrated CX_3_CL1 protein expression from reactive type II pneumocytes, airway epithelial cells, and epithelial cells involved in bronchiolization as well as interstitial infiltrating mononuclear cells (Fig 2A–2D). There was no significant vascular or peri-vascular staining of CX_3_CL1 from or surrounding vessels with or without a vasculopathy.

Alternatively, CX_3_CR1 was only expressed from infiltrating interstitial mononuclear cells. Morphometrically we identified many of these cells as plasma cells by their specific cartwheel nuclei shape that is eccentrically located (Fig 2E and 2F) within the cytoplasm. We confirmed that these were plasma cells using their cell surface marker CD138 (Fig 2G). Furthermore, using dual immunofluorescence (IF) staining we demonstrate that the plasma cell (red CD138) are localized with CX3CR1 (green) (Fig 2H).

### OUH SSc cohort demographic and clinical characteristics and associations with CX_3_CL1

Complete data were available in 292 SSc cases. RHC was conducted in 92 (31.5%) of the SSc patients. The total SSc cohort age was 48.1 years (SD 8.1), 53 (18.2%) were males, 275 (94.2%) were Caucasian, 2 (0.7%) Black, and 15 (5.1%) were Asian (Table 1). Seventy seven (26.4%) had dcSSc, 47 (16.1%) were ATA positive and 215 (73.6%) had esophageal dysmotility (Table 1). In total, 63 (21.6%) had received at least one immune modulating treatment; 51 (17.5%) started with treatment at some point during the follow up period.

We found significantly increased concentrations of CX_3_CL1 from the serum of the SSc patients (2.0 ng/ml (SD 2.5)) as compared to the healthy controls (1.1 ng/ml (SD 1.4), p = 0.002) (Fig 1B). CX_3_CL1 was associated with ATA (OR 1.2, 95% CI 1.10–1.42, p = 0.001) (Fig 1C). There were no significant associations with male gender, dcSSc, mortality, digital ulcers, scleroderma renal crisis, gastrointestinal symptoms,the mRSS or use of immune modulating medication.

### Serum concentration of CX_3_CL1 is associated with baseline presence of ILD, severity of ILD, but not PH

The cohort included SSc patients with and without pulmonary manifestations such as PH and ILD (Table 2). At baseline, 36 (12.3%) SSc patients had RHC verified PH, 51 (17.5%) had clinically significant ILD (>10% pulmonary fibrosis on HRCT), 117 (40%) mild ILD with 0.1–10% pulmonary fibrosis and 124 patients (42.5%) had no sign of pulmonary fibrosis.

**Table 2:** CX_3_CL1 concentrations are associated with baseline cardiopulmonary characteristics by multivariable logistic regression analyses.

	No (%)	OR (95% CI)	P-Value
Pulmonary fibrosis ≥10%	51 (17.5)	1.13 (1.01–1.27)	0.044
FVC <70%	37 (12.7)	1.06 (0.94–1.02)	0.359
DLCO <60%	103 (35.3)	1.16 (1.03–1.30)	0.014
PH	36 (12.3)	1.00 (0.86–1.17)	0.968
sPAP ≥30 mmHg on ECHO	99 (33.9)	1.01 (0.90–1.10)	0.781
NT-proBNP ≥165 pg/mL	24 (8.2)	1.10 (0.96–1.27)	0.159

OR: Odds ratio; CI: confidential interval; FVC: forced vital capacity; DLCO: diffusing Lung capacity for carbon monoxide; PH: pulmonary hypertension; PAH: pulmonary arterial hypertension; ILD: interstitial lung disease; sPAP: systolic pulmonary arterial pressure; ECHO: echocardiography; NT-proBNP: N-terminal pro-brain natriuretic peptide

We found significant correlations between serum concentrations of CX_3_CL1 and extent of baseline fibrosis on HRCT (r = 0.2, p = 0.039), as well as significant lung fibrosis >10% (Table 2, Fig 1D). DLCO <60% at baseline was also significantly associated with CX_3_CL1, but there were no significant associations with baseline FVC (Table 2). Importantly, these results did not significantly change after exclusion of patients with PAH at baseline (data not shown). Conversely, we found no association between serum CX_3_CL1 concentrations and baseline PH including WHO group 1 PAH, and WHO group 3 PH-ILD, sPAP on ECHO, or NT-proBNP (Table 2).

### Elevated serum concentrations of CX_3_CL1 correlate with SSc-ILD progression

In total, 212 (72.6%) SSc patients met the primary ILD progression free survival composite endpoint while 80 (27.4%) developed an ILD progression event or died (Table 3, S1 Fig). CX_3_CL1 was in univariable analyses significantly associated with the primary endpoint ILD progression (OR 1.24, 95% CI 1.08–1.41, p = 0.002), as well as age at onset (OR 1.05, 95% CI 1.03–1.07, p<0.001), mRSS (OR 1.03, 95% CI 1.00–1.07, p = 0.039), and baseline DLCO% (OR 0.98, 95% CI 0.97–0.99, p = 0.025).

**Table 3:** CX_3_CL1 association with the primary and secondary endpoints by univariable logistic regression analysis.

	Event No (%)	OR (95% CI)	P-Value
**Primary ILD related endpoints**			
Composite ILD endpoint*	80 (27.4)	1.24 (1.08–1.41)	0.002
Annual FVC decline ≥5%	51 (17.5)	1.08 (0.95–1.24)	0.242
Annual DLCO decline ≥7.5%	29 (9.9)	1.34 (1.15–1.56)	<0.001
Deceased	24 (8.2)	0.97 (0.87–1.08)	0.843
**Secondary ILD related endpoints**			
New onset significant ILD on HRCT^†^	20 (6.8)	1.33 (1.04–1.70)	0.025
Annual progression of pulmonary fibrosis >5%	9 (3.1)	1.06 (0.31–1.44)	0.302

OR: Odds ratio; CI: confidential interval

*Composite ILD endpoint: FVC decline ≥5%, or DLCO decline ≥7.5%, or death within 12 months; ILD: interstitial lung disease; FVC: forced vital capacity; DLCO: diffusing Lung capacity for carbon monoxide

^†^progression of lung fibrosis from < 10% to >10% fibrosis on HRCT

In multivariable analyses, CX_3_CL1 was also associated with ILD progression (AUC = 0.82) shown in Table 4. Results from the univariable analyses are shown in the S1 Table.

**Table 4:** Multivariable logistic regression analyses with the primary composite ILD endpoint including FVC decline ≥5%, or DLCO decline ≥7.5%, or death within 12 months in the Oslo University Hospital SSc cohort.

	OR (95%CI)	p-value
CX_3_CL1, pg/μl	1.30 (1.12–1.52)	0.001
Age at onset, yrs	1.07 (1.04–1.10)	<0.001
mRSS	1.04 (1.00–1.08)	0.050
Male sex	1.41 (0.59–4.38)	0.437

OR: Odds ratio; CI: confidential interval; DLCO: diffusing lung capacity for carbon monoxide; sPAP: systolic pulmonary arterial pressure on ECHO; mRSS: modified Rodnan Skin Score, area under the roc-curve (AUC): 0.80.

Analysis of the secondary endpoints demonstrated, that CX_3_CL1 in univariable analyses associates with new onset of significant lung fibrosis (progressed from <10% lung fibrosis to >10%) (Table 3). In multivariable analyses, CX_3_CL1 (OR 1.4, 95% CI 1.06–1.84, p = 0.016) and the extent of baseline fibrosis (OR 1.5, 95% CI 1.26–1.74), p<0.001) were associated with new onset of significant lung fibrosis (AUC = 0.87). CX_3_CL1 was associated with annual progression of pulmonary fibrosis > 5% in univariable analyses (Table 3), no further analyses were performed due to low events (n = 9). Similar results were found when we excluded patients with PAH at baseline (data not shown).

Twenty nine SSc patients (9.9%) developed PH during the observation period and did not meet the PH free endpoint (Table 5). Of those, 18 patients (6.2%) were diagnosed with WHO group 1 PAH and 11 (3.8%) with WHO group 3 (PH-ILD). Analysis of the primary and secondary endpoints showed that CX_3_CL1 was not significant associated with new onset of PH, sPAP>30mmHg on ECHO, increase of sPAP on ECHO by ≥10 mmHg or NT-proBNP≥ 165 pg/ml (Table 5).

**Table 5:** Frequency and univariable logistic regression analyses of CX_3_CL1 and new events of primary and secondary PH endpoints including during the observation period in the Oslo University Hospital SSc cohort.

	Event No (%)	OR (95% CI)	P-Value
**Primary endpoints**			
PH development	29 (9.9)	1.06 (0.94–1.20)	0.354
PAH	18 (6.2)	1.08 (0.93–1.25)	0.323
PH-ILD	11 (3.8)	1.03 (0.84–1.27)	0.764
**Secondary PH related endpoints**			
sPAP ≥30 mmHg on ECHO	50 (17.1)	1.04 (0.94–1.16)	0.412
Increase of sPAP on ECHO by ≥10 mmHg	92 (31.5)	1.06 (0.96–1.17)	0.236
NT-proBNP ≥165 pg/mL	26 (8.9)	0.97 (0.82–1.15)	0.750

OR: Odds ratio; CI: confidential interval; PH: pulmonary hypertension; PAH: pulmonary arterial hypertension; sPAP: systolic pulmonary arterial pressure; ECHO: echocardiography; NT-proBNP: N-terminal pro-brain natriuretic peptide

## Discussion

SSc carries high risk for PH and ILD as well as their progression to right heart failure and end stage pulmonary fibrosis; respectively. However, little is known about the pathogenesis of these associated diseases in SSc and there is a paucity of data demonstrating ways to stratify pulmonary disease risk in SSc. Here, we examined the role of the CX_3_CR1/CX_3_CL1 biological axis in the pathogenesis of SSc associated lung disease. We demonstrate that CX_3_CL1 is associated with baseline severity of ILD and progression of ILD, and identified CX_3_CR1 expressing plasma cells as a novel recruited interstitial lung effector cell population in SSc-ILD, suggesting it may contribute to the pathobiology of SSc-ILD.

To our knowledge, this is the largest cohort assessing the role of CX_3_CL1 in SSc associated ILD and PH and the only study including two independent cohorts with both lung homogenate and sera analysis of CX_3_CL1 concentrations. In this study, all patients had longitudinal data on extent of fibrosis on HRCT and PFTs available. CX_3_CL1 was associated with extent of fibrosis at baseline and progressive ILD including lung fibrosis and DLCO decline. There have been few other studies on CX_3_CL1 in SSc cohorts and the results have been conflicting. In 2005, *Hasegawa et al* showed in a cohort including 67 SSc patients that CX_3_CL1 was expressed on endothelial cells in SSc skin and lung tissue. In line with our data, they found that circulating CX_3_CL1 was associated with the severity of pulmonary fibrosis evaluated on chest x-ray, and not with PH [27]. Conversely, *De Lauretis et al* did not find associations between CX_3_CL1 and short term lung function deterioration in a recent study involving 74 SSc patients [28].

We believe that our study involving lungs and sera from SSc patients, that consists of greater than double the numbers of patients from the above two smaller studies combined, may settle the controversy between the association of CX_3_CL1 and SSc-ILD, as compared to SSc-PH. Importantly, our study also demonstrates that elevated concentrations of CX_3_CL1 is associated with ILD progression. Thus, if validated this would advocate that CX_3_CL1 concentrations are a biomarker that when elevated would suggest the clinician could initiate ILD directed therapy, which could prevent a further decline in lung function from progressive SSc-ILD.

Several authors have described potential roles of the CX_3_CL1/CX_3_CR1 axis in the development of pulmonary vascular disease in SSc [12,29,30]. Interestingly, we did not find any correlation to RHC verified PH, sPAP on ECHO or NT-proBNP in the two SSc cohorts and no CX_3_CL1/CX_3_CR1 protein expression on endothelial cells strengthening the assumption that CX_3_CL1 is associated with SSc-ILD and not pulmonary vascular disease.

Mechanistically, the current study supports the notion that epithelial cells and not endothelial cells are producing CX_3_CL1. More specifically, CX_3_CL1 is produced by type 2 pneumocytes, airway epithelium and epithelial cells involved in bronchiolization of honeycomb cysts. CX_3_CR1 is only found on infiltrating interstitial mononuclear cells, but not perivascular mononuclear cells. Importantly, of all the infiltrating interstitial mononuclear cells, CX_3_CR1 is predominately localized to plasma cells. Interestingly, we found that CX_3_CL1 concentrations from the lung and sera correlate with ATA antibodies. Collectively, this implies that the CX_3_CR1/ CX_3_CL1 biological axis is involved in the recruitment of plasma cells to the interstitium that are generating autoantibodies that eventually leads to the damage of the parenchyma of SSc-ILD lungs. In corroboration, we also found that increased concentrations of CX_3_CL1 correlates inversely with DLCO in both the SSc-lungs from the UCLA cohort and the sera of the OUH cohort. Collectively, our data demonstrating that CX_3_CL1 has no association to PH, suggests that the CX_3_CR1/CX_3_CL1 biological axis is, in part, contributing to the pathobiology of SSc-ILD.

We believe that the current study has major strengths. Firstly, CX_3_CL1 was assessed in two independent cohorts corroborating the results including two different but complementary sources of biomaterial. Secondly, CX_3_CL1 levels were examined in sera in a large and unselected cohort with longitudinal follow-up data including complete lung follow up data with function tests, lung fibrosis as assessed by HRCT, ECHO as well as RHC data resulting in reliable data on clinical associations to CX_3_CL1. Thirdly, CX_3_CL1 protein was assessed in 12 patients at time of transplant and cellular sources of CX_3_CL1 and CX_3_CR1 were demonstrated by IHC giving insights in the pathobiology of CX_3_CL1/CX_3_CR1.

There are some limitations. CX_3_CL1 was measured only cross sectional. Additionally, all lung homogenates were end stage samples since they were collected at time of transplant. Also, the inter-individual variations in disease duration and the variation in observational length are a limitation. This is due to the OUH SSc study design where patients are included consecutively and then followed longitudinally. Since the OUH cohort is population-based, and covers the whole spectrum of disease, the frequency of SSc cases with progressing lung disease is lower than in selected cohorts. Since we only included patients with baseline and follow up HRCT scans, it selected against patients who died early in the disease course and survival bias was an unavoidable limitation [14,15]. This may explain why we did not find associations between augmented CX_3_CL1 levels and reduced survival.

In conclusion, we have demonstrated an association between elevated protein levels of CX_3_CL1 and progressive SSc-ILD and have shown that within the lungs, CX_3_CL1 is predominately localized with epithelia and its receptor, CX_3_CR1 to infiltrating interstitial mononuclear cells. These results suggest that CX_3_CR1/CX_3_CL1 biological axis may be a driver of dysregulated autoimmunity that favours SSc-ILD and may have an ability to predict SSc-ILD as well as its progression.

## Supporting information

S1 Table Univariable logistic regression analyses of the composite ILD endpoint. Univariable logistic regression analyses of clinical characteristics and the composite ILD outcome measure in the Oslo University Hospital SSc cohort.
(PDF)

S1 Fig Venn diagram for the primary ILD endpoint. Venn diagram including the frequency of events for each parameter included in the primary composite ILD outcome; with the blue circle showing the frequency of annual FVC decline >5%, the red circle showing the frequency of annual DLCO decline>7.5% and the green circle showing the frequency of the 1-year mortality.
(PDF)

S1 File Anonymized data from the OUH cohort. Anonymized data file including clinical data and CX_3_CL1 levels SSc patients and healthy controls.
(ZIP)

S2 File Anonymized data from the UCLA cohort. Anonymized data file including clinical data and CX_3_CL1 levels SSc patients and healthy controls.
(ZIP)
