# Supplementary material for: Augmented concentrations of CX3CL1 are associated with interstitial lung disease in systemic sclerosis
Source: PLoS One. 2018 Nov 20;13(11):e0206545. doi: 10.1371/journal.pone.0206545 (PMC6245508; doi:10.1371/journal.pone.0206545)
Supplement: S1 Table — Univariable logistic regression analyses of clinical characteristics and the composite ILD outcome measure in the Oslo University Hospital SSc cohort. (PDF) [file pone.0206545.s001.pdf]

Supplementary information

S1 Table. Univariable logistic regression analyses for the composite ILD endpoint

Univariable logistic regression analyses of clinical characteristics and the composite ILD outcome measure in the Oslo University Hospital SSc cohort

	OR (95% CI)	P-Value
Age at disease onset	1.05 (1.03-1.07)	<0.001
Male sex	1.14 (0.57-2.29)	0.701
Smoker	1.09 (0.60-1.85)	0.843
Diffuse cutaneous SSc	1.03 (0.56-1.87)	0.927
ATA	1.08 (0.60-1.94)	0.798
ACA	0.71 (0.41-1.21)	0.201
Modified Rodnan Skin Score	1.03 (1.00-1.07)	0.039
Baseline fibrosis	1.02 (0.99-1.05)	0.089
Baseline FVC	0.99 (0.98-1.08)	0.443
Baseline DLCO	0.99 (0.97-0.99)	0.025
Digital ulcers	0.82 (0.41-1.64)	0.573
Esophagus dysmotiliy	0.66 (0.34-1.29)	0.219
Dysphagia	0.98 (0.51-1.72)	0.952
Scleroderma renal crisis	1.14 (0.27-4.94)	0.856
GAVE	1.33 (0.51-3.44)	0.552
Immune modulating treatment	0.79 (0.39-1.60)	0.508

OR: odds ratio; CI: confidence interval; SSc: systemic sclerosis; ATA: anti-topoisomerase I antibody; ACA: anti-centromere antibody; FVC: forced vital capacity; DLCO: diffusing lung capacity for carbon monoxide; GAVE: Gastric Antral Vascular Ectasia
