# Supplementary material for: Augmented concentrations of CX3CL1 are associated with interstitial lung disease in systemic sclerosis
Source: PLoS One. 2018 Nov 20;13(11):e0206545. doi: 10.1371/journal.pone.0206545 (PMC6245508; doi:10.1371/journal.pone.0206545)

S1 Fig: Venn diagram for the primary ILD endpoint

Venn diagram including the frequency of events for each parameter included in the primary composite ILD outcome; with the blue circle showing the frequency of annual FVC decline >5%, the red circle showing the frequency of annual DLCO decline>7.5% and the green circle showing the frequency of the 1-year mortality

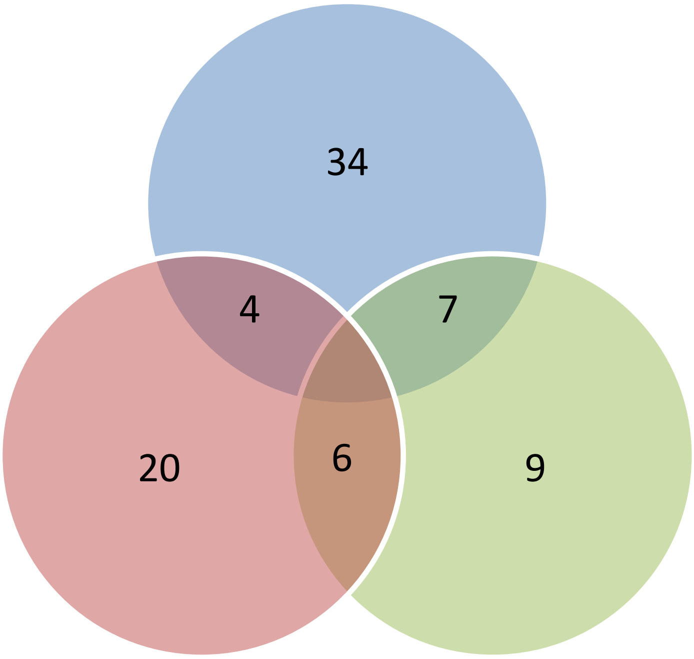

Supplement: S1 Fig — Venn diagram including the frequency of events for each parameter included in the primary composite ILD outcome; with the blue circle showing the frequency of annual FVC decline >5%, the red circle showing the frequency of annual DLCO decline>7.5% and the green circle showing the frequency of the 1-year mortality. (PDF) [file pone.0206545.s002.pdf]
